# CYP2C19 polymorphisms and lipoproteins associated with clopidogrel resistance in children with Kawasaki disease in China: A prospective study

**DOI:** 10.3389/fcvm.2022.925518

**Published:** 2022-08-22

**Authors:** Mingming Zhang, Li Meng, Yeshi Chen, Xiaohui Li, Lin Shi

**Affiliations:** ^1^Department of Cardiology, Children’s Hospital Capital Institute of Pediatrics, Beijing, China; ^2^Capital Institute of Pediatrics-Peking University Teaching Hospital, Beijing, China

**Keywords:** Kawasaki disease, clopidogrel resistance, lipoproteins, CYP2C19 polymorphisms, risk factor

## Abstract

**Background:**

CYP2C19 genetic variation and clinical factors have been proved to be related with clopidogrel resistance (CR) in adults, while the presence of CR in children with Kawasaki disease (KD) was seldom reported. Our objective was to evaluate KD patients’ response to clopidogrel treatment and determine whether CYP2C19 gene polymorphisms and laboratory indicators are associated with CR in this population.

**Methods:**

This was a prospective and single-center study. We recruited children with KD hospitalized in the cardiology department at the Children’s Hospital Capital Institute of Pediatrics between January 2019 and October 2021, and the distribution of the CYP2C19 gene polymorphisms was assessed. According to the light transmission aggregometry (LTA) test results, KD patients who were treated with clopidogrel were divided into CR group and non-CR (NCR) group. We also analyzed the influence of CYP2C19 gene polymorphisms and laboratory indicators on CR in children with KD.

**Results:**

(1) A total of 346 children with KD were evaluated for the genotypic and phenotypic distributions of CYP2C19. Loss-of-function (LOF) mutated allele was included in 56.9% of CYP2C19 genotypes, and their corresponding phenotypes were intermediate metabolizers (46.2%) and poor metabolizers (10.7%). (2) The incidence of CR in this study population was 31.4%. The multivariate logistic regression showed that carrying CYP2C19 LOF allele (OR, 3.922; 95%CI, 1.504–10.282; *P* = 0.005) and high levels of low-density lipoprotein (OR, 1.675; 95%CI, 1.069–2.623; *P* = 0.024) were independent risk factor for CR, while low levels of high-density lipoprotein (OR, 0.120; 95%CI, 0.020, 0.734; *P* = 0.022) was an independent protective factor for CR. The area under the receiver operator characteristic curve of the multivariate logistic regression model (including high-density lipoprotein, low-density lipoprotein, and CYP2C19 LOF allele carriers) for predicting CR was 0.769 (95% CI, 0.674–0.863; *P* < 0.001). The sensitivity and specificity were 70.3 and 74.0%, respectively.

**Conclusion:**

Carrying CYP2C19 LOF allele, low levels of high-density lipoprotein, and high levels of low-density lipoprotein were independent risk factors for CR in children with KD in China. This may benefit pediatricians in choosing appropriate individualized antiplatelet therapy.

## Introduction

Kawasaki disease (KD) is an acute systemic vasculitis that occurs predominantly in children younger than 5 years old. Coronary artery thrombosis (CAT) is the most severe complication of KD, placing patients at risk for coronary artery occlusion or stenosis, myocardial infarction, and even sudden cardiac death (1, 2). Given that thrombocytosis and platelet activation are high-risk factors for CAT, antiplatelet therapy has become a mainstay in the prevention of CAT in KD (3). Antiplatelet agents recommended by current guidelines for KD include aspirin, dipyridamole, and clopidogrel (3, 4). Although aspirin is the first-line antiplatelet treatment of KD, some children with KD need to replace or combine it with other antiplatelet agents due to intolerance or resistance to aspirin (2). Additionally, dipyridamole may lead to “coronary artery steal” and further progression of myocardial ischemia (4). Based on the above reasons, clopidogrel is increasingly used in KD patients. However, how to optimally apply clopidogrel in children with KD has become a practical problem.

Clopidogrel is an adenosine diphosphate (ADP) receptor P2Y12 antagonist, which can effectively inhibit platelet aggregation. Many large clinical trials have identified the efficacy and safety of clopidogrel in adults with cardiovascular and cerebrovascular diseases (5–8). At the same time, studies have confirmed that there is substantial interpatient variability in the response to clopidogrel, some patients still experience thrombosis following long-term oral clopidogrel therapy (9). This failure of inhibiting platelet aggregation in clopidogrel users refers to clopidogrel resistance (CR) in adults (10). Although the mechanism of CR has not been fully elucidated in adults, existing studies have established its relationship with genetic and clinical factors (10–12). On the one hand, some studies showed that CYP2C19 gene polymorphisms can be used as predictors of CR in adults (13). Among them, carrying the CYP2C19 loss-of-function (LOF) alleles (especially the most common CYP2C19 *2 and CYP2C19 *3 variants) is associated with insufficient inhibition of platelet activity or increased risk of CR (14–16). Simultaneously, carrying the CYP2C19 gain-of-function (GOF) allele (CYP2C19 *17) contributes to the antiplatelet effect of clopidogrel and may be associated with increased risk of bleeding complications (17). On the other hand, clinical factors such as clopidogrel dosage, drug interaction (e.g., statins), platelet function, liver metabolic function, and renal dysfunction can also affect the antiplatelet effect of clopidogrel (10, 13, 18–20). In pediatric population, the clinical application of clopidogrel is limited, and it is mainly used for pediatric cardiac conditions, such as congenital heart disease, intracardiac stents or devices, and KD (21, 22). Likewise, published information about the efficacy and safety of clopidogrel in pediatric diseases such as KD is very insufficient, and the currently available literatures are mainly case series reports and small retrospective studies, lacking evidence-based medical data from prospective multicenter randomized controlled trials (21, 22). Besides, children’s physiological characteristics and disease states are different from adults’, so it remains uncertain whether the findings in adults are applicable to children. To provide a better basis for the application of clopidogrel in KD children, this study focused on KD children and assessed the distribution of the CYP2C19 gene polymorphisms (*2, *3, *17) in them. Then, we evaluated the antiplatelet effect of clopidogrel in children with KD and further analyzed the impact of CYP2C19 gene polymorphisms (especially LOF alleles) and laboratory indicators on CR.

## Materials and methods

### Patients and study design

This study was a prospective study in a single center. We recruited children with KD hospitalized in the cardiology department at the Children’s Hospital Capital Institute of Pediatrics between January 2019 and October 2021.

The inclusion criteria were as follow: (1) patients who were first diagnosed with KD based on the guidelines of the American Heart Association (AHA) published in 2017 (3); (2) patients who signed the informed consent form to receive clopidogrel and completed the detection of platelet aggregation function, CYP2C19 genotype (*2, *3, *17 alleles) and related laboratory indicators during hospitalization.

The exclusion criteria were as follow: (1) Comorbidity with serious dysfunction of bleeding and blood coagulation; (2) contraindications to clopidogrel (e.g., severe liver damage, active bleeding, drug allergy); (3) incomplete clinical data; (4) patients who could not cooperate with the research or withdraw informed consent.

According to the light transmission aggregometry (LTA) test results, KD patients treated with clopidogrel were divided into CR group and non-CR (NCR) group. CR was defined as ADP-induced platelet aggregation rate (ADP%) ≥ 50% at a concentration of 5 μmol/L, and NCR was ADP% < 50% (23).

The study was approved by the Ethics Committee of the Capital Institute of Pediatrics (No. SHERLL 2018020). Informed consent was obtained from a parent or guardian of each patient before the study.

### Platelet function measurement

Peripheral blood was taken from each participant into citrate anticoagulant tube on an early morning fasting after 3–5 days of the clopidogrel treatment. After inversion and mixing for 3 times, it was immediately sent to the laboratory for testing (Agg-RAM platelet aggregator, Helena company, United States), and the inducer was ADP (5 μmol/L). The contact between blood collector and glassware was strictly avoided during blood draw.

### CYP2C19 genotyping and phenotyping

From each patient, we acquired a 2 ml sample of peripheral blood in an EDTA anticoagulant tube following early morning fasting before the treatment. Serum was separated by centrifugation and 200–500 μl of blood cells were stored in a refrigerator at –80°C. DNA was extracted from blood cells for genotyping according to the Genomic DNA extraction kit instructions. Genotyping of the CYP2C19 (*1,*2,*3,*17) was performed using the Sequenom MassARRAY iPLEX platform (Sequenom, San Diego, CA). The locus–specific PCR primers were designed by the MassARRAY Assay Design software package (v4.0). The PCR products were dephosphorylated with Shrimp Alkaline Phosphatase enzymes before undertaking the iPLE X GOLD primer extension reactions. The desalted iPLEX reaction product was spotted onto a 384-format SpectroCHIP. Mass determination was discriminated by the MALDI-TOF mass spectrometer. Data acquisition was done by the MassARRAY Typer 4.0 software.

According to clinical guidelines issued by the Clinical Pharmacogenetics Implementation Consortium (CPIC) (16), the CYP2C19 alleles were categorized into functional groups as follows: normal function allele (CYP2C19 *1), LOF allele (CYP2C19 *2, CYP2C19 *3), and GOF allele (CYP2C19 *17). The combination of alleles determined the genotypes (also referred to as diplotypes), which included the wild genotype (*1/*1) and the mutated genotype. CYP2C19 LOF allele carriers were defined as patients with at least one LOF allele (*1/*2, *1/*3, *2/*2, *2/*3, *3/*3, *2/*17, or *3/*17). The CYP2C19 phenotypes were defined by allele function combinations and were grouped into the following 5 predicted metabolic phenotypes: (1) Normal metabolizers (NMs): Patients carried two normal function alleles (*1/*1); (2) intermediate metabolizers (IMs): patients carried one normal function allele and one LOF allele (*1/*2,*1/*3) or carried one LOF allele and one GOF allele (*2/*17,*3/*17); (3) poor metabolizers (PMs): patients carried two LOF alleles (*2/*2, *2/*3, *3/*3); (4) rapid metabolizers (RMs): patients carried one normal function allele and one GOF allele (*1/*17); (5) ultrarapid metabolizers (UMs): patients carried two GOF alleles (*17/*17).

### Clinical data

We collected a range of clinical data, including age, sex, clopidogrel dosage, and the following laboratory indicators before clopidogrel treatment: (1) Platelet-related indicators: platelet (PLT), mean platelet volume (MPV), platelet distribution width (PDW), plateletcrit (PCT); (2) Liver metabolism indicators: Alanine aminotransferase (ALT), aspartate aminotransferase (AST), albumin (ALB), high-density lipoprotein (HDL), low-density lipoprotein (LDL), triglyceride (TG); (3) Renal function indicators: Blood urea nitrogen (BUN) and serum creatinine (SCr). If these laboratory indicators were performed several times before treatment, the lowest value was adopted for HDL and ALB, while the highest value was adopted for the remaining indicators.

### Statistical analysis

All analyses were conducted by SPSS Version 26.0. *P* < 0.05 was considered statistically significant (two-tailed). For continuous variables, Kolmogorov-Smirnov test was carried out to test for normality of the distribution. Non-normally distributed continuous variables were presented as median and interquartile range (IQR, P25-P75), and Mann-Whitney *U*-test was used for comparing two groups. Categorical variables were presented as frequency and percentages (%) in each category, and Pearson’s chi-squared test was used to compare between groups. Multivariable logistic regression analysis was performed using stepwise logistic regression with forward selection and backward elimination by removing variables that had a *p*-value greater than 0.05. Results were expressed as odds ratio (OR) with 95% confidence interval (CI). Finally, to evaluate the markers’ ability to predict CR, analysis of the receiver operating characteristics curve (ROC) by the area under the curve (AUC) was performed according to logistic regression.

## Results

### Demographic data

**FIGURE 1:**
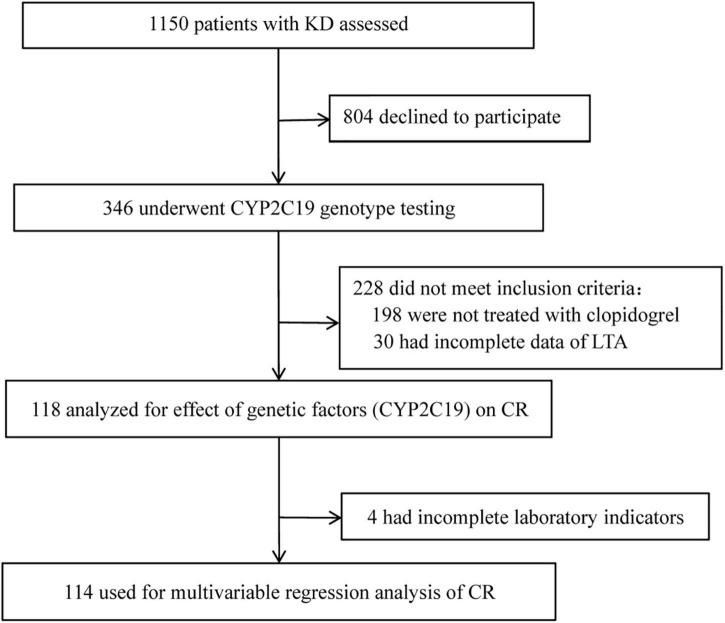
Flow diagram of the study. KD, Kawasaki disease; LTA, light transmission aggregometry; CR, clopidogrel resistance.

A flow diagram of the study is shown in Figure 1. A total of 346 patients were successfully genotyped for CYP2C19 to assess the genotypic and phenotypic distributions of the CYP2C19: 209 males and 137 females (male/female = 1.53:1) with the median age of 2.00 years old (IOR, 1.08–3.50 years). Of the 346 cases, 114 cases were included in the multivariable regression analysis of CR in children with KD: 75 males and 39 females (male/female = 1.92:1), with a median age of 1.67 years old (IOR, 0.96–3.00 years). In this study, 8.57% (10/118) of children with KD were found to have minor bleeding events during oral clopidogrel, including 4 cases of mild epistaxis and 6 cases of skin ecchymosis. No serious bleeding events such as gastrointestinal bleeding was found.

### Distribution of CYP2C19 gene polymorphisms in children with Kawasaki disease

Regarding CYP2C19 in 346 KD patients, the distribution of CYP2C19 alleles was 64.5% for normal allele, 33.8% for LOF allele, and 1.7% for GOF allele. The CYP2C19 genotypes included 41.3% (143/346) wild genotype and 58.7% (203/346) mutated genotype, and the rate of LOF mutated allele carriers was 56.9% (197/346). According to the different genotypes of CYP2C19, they were divided into 4 metabolic phenotypes: NMs (41.3%), RMs (1.7%), IMs (46.2%), and PMs (10.7%) (Table 1). Patient of UMs type was not found in this study.

**TABLE 1:** Distribution of CYP2C19 gene polymorphisms in children with KD.

Gene	Allele	Frequency	Genotype	Distribution	Phenotype	Distribution
CYP2C19	*1	446 (64.5%)	*1/*1	143 (41.3%)	NMs	143 (41.3%)
	*2	202 (29.2%)	*1/*17	6 (1.7%)	RMs	6 (1.7%)
	*3	32 (4.6%)	*1/*2	132 (38.2%)	IMs	160 (46.2%)
	*17	12 (1.7%)	**3	22 (6.4%)		
			*2/*17	5 (1.4%)		
			*3/*17	1 (0.3%)		
			*2/*2	29 (8.4%)	PMs	37 (10.7%)
			*2/*3	7 (2.0%)		
			*3/*3	1 (0.3%)		

KD, Kawasaki disease; NMs, normal metabolizers; RMs, rapid metabolizers; IMs, intermediate metabolizers; PMs, poor metabolizers.

### Factors associated with the efficacy of clopidogrel in children with Kawasaki disease

#### Gene polymorphisms of CYP2C19

According to the results of LTA, 118 children with KD who received clopidogrel were divided into the CR group and NCR group, of which the CR group accounted for 31.4% (37/118). There were no statistically significant differences in the baseline characteristics between the two groups in terms of sex, age and clopidogrel dosage (*P* > 0.05). Tests of CYP2C19 gene polymorphisms showed that the proportion of CYP2C19 LOF allele carriers in the CR group was significantly higher than that in the NCR group (78.4 vs. 45.7%, *P* = 0.001) (Table 2).

**TABLE 2:** Comparison of CYP2C19 gene polymorphisms in CR group and NCR group.

Variables	CR-group (*n* = 37)	NCR-group (*n* = 81)	*P*-value
Age, years, median (IQR)	1.67 (0.88–2.92)	1.67 (0.92–3.04)	0.956
Sex (male), n (%)	26 (70.3%)	53 (65.4%)	0.604
Clopidogrel dose, median (IQR), (mg/kg/d)	0.78 (0.69–0.93)	0.81 (0.68–0.99)	0.435
CYP2C19 LOF allele carriers*, n (%)	29 (78.4%)	37 (45.7%)	**0.001**

CR, Clopidogrel resistance; NCR, No clopidogrel resistance; IQR, interquartile range, P25-P75; LOF, loss-of-function.

*CYP2C19 LOF allele carriers were defined as patients with at least one LoF allele (*1/*2, *1/*3, *2/*2, *2/*3, *3/*3, *2/*17, or *3/*17). Bold values represent P values less than 0.05.

#### Laboratory indicators

After excluding the 4 children without laboratory indicators, 114 children were divided into the CR group and NCR group to analyze the effect of laboratory indicators on the efficacy of clopidogrel in children with KD. There were no statistically significant differences in the baseline characteristics between two groups in terms of sex, age and clopidogrel dosage (*P* > 0.05). The results showed that HDL and LDL were statistically different between the two groups (*P* < 0.05). Levels of HDL [0.65 (0.50–0.84) vs. 0.77 (0.64–1.01) mmol/L, *P* = 0.005] were significantly lower, and levels of LDL [3.00 (2.27–3.46) vs. 2.51 (1.98–3.10) mmol/L, *P* = 0.025] were significantly higher in the CR group than in the NCR group. No statistically significant difference was found in other examined laboratory indicators such as the levels of PLT, MPV, PDW, PCT, ALT, AST, ALB, BUN, and SCr between the two groups (Table 3).

**TABLE 3:** Comparison of laboratory indicators in CR group and NCR group.

Variables	CR-group (*n* = 37)	NCR-group (*n* = 77)	*P*-values
Age, years, median (IQR)	1.67 (0.88–2.92)	1.67 (0.92–3.00)	0.930
Sex (male), n (%)	26 (70.3%)	49 (63.6%)	0.485
Clopidogrel dose, median (IQR), (mg/kg/d)	0.78 (0.69–0.93)	0.81 (0.68–0.96)	0.569
PLT, median (IQR), × 10^9^/L	601.00 (448.00–709.00)	518.00 (413.00–648.00)	0.150
MPV, median (IQR), fL	9.20 (8.70–9.80)	9.20 (8.70–9.70)	0.983
PDW, median (IQR), fL	9.10 (8.50–10.10)	9.10 (8.40–10.30)	0.981
PCT, median (IQR),%	0.56 (0.44–0.62)	0.48 (0.40–0.61)	0.078
ALT, median (IQR), U/L	30.40 (18.95–103.85)	21.80 (13.90–40.20)	0.228
AST, median (IQR), U/L	33.20 (20.95–52.90)	33.70 (22.65–51.85)	0.966
ALB, median (IQR), g/L	36.80 (33.75–39.90)	36.50 (32.85–40.75)	0.672
HDL, median (IQR), mmol/L	0.65 (0.50–0.84)	0.77 (0.64–1.01)	**0.005**
LDL, median (IQR), mmol/L	3.00 (2.27–3.46)	2.51 (1.98–3.10)	**0.025**
TG, median (IQR),mmol/L	1.41 (1.06–2.34)	1.44 (0.95–2.05)	0.543
BUN, median (IQR), mmol/L	3.60 (2.53–4.63)	3.39 (2.17–5.66)	0.670
SCr, median (IQR), μmol/L	22.60 (19.45–26.10)	22.80 (22.30–26.10)	0.596

CR, clopidogrel resistance; NCR, non-clopidogrel resistance; IQR, interquartile range, P25-P75; PLT, platelet; MPV, Mean platelet volume; PDW, Platelet distribution width; PCT, plateletcrit; ALT, alanine aminotransferase; AST, aspartate aminotransferase; ALB, albumin; HDL, high-density lipoprotein; LDL, low-density lipoprotein; TG, Triglyceride; BUN, blood urea nitrogen; SCr, serum creatinine. Bold values represent P values less than 0.05.

### Multivariable logistic regression and receiver operating characteristics curve analysis for clopidogrel resistance in children with Kawasaki disease

**FIGURE 2:**
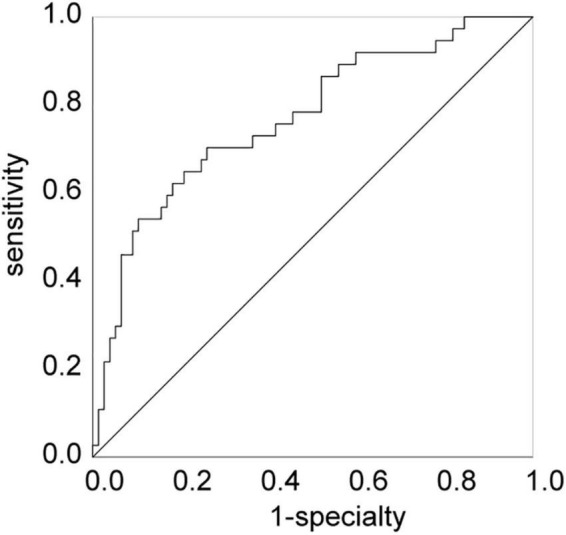
The ROC curve of the multivariable logistic regression model. ROC, receiver operating characteristics curve.

The results revealed carrying CYP2C19 LOF allele and high levels of LDL were independent risk factor for CR (OR > 1, *P* < 0.05), while low levels of HDL (OR < 1, *P* < 0.05) was an independent protective factor for CR (Table 4). The validity of these factors in predicting CR in patients with KD was assessed using ROC curve analysis. The AUC of the various variables for predicting CR were 0.664, 0.630, and 0.671 for HDL, LDL, and carrying the CYP2C19 LOF allele, respectively. The HDL cut-off value of 0.70 mmol/L, LDL cut-off value of 2.92 mmol/L, and carrying the CYP2C19 LOF allele yielded sensitivities of 64.9, 62.2, and 78.4%, and specificities of 67.5, 68.8, and 55.8% for predicting CR, respectively (Table 5). Besides, the AUC of the multivariate logistic regression model (including HDL, LDL, and CYP2C19 LOF allele carriers) for predicting CR was 0.769. The sensitivity and specificity were 70.3 and 74.0%, respectively (Figure 2).

**TABLE 4:** Multivariable logistic regression analysis for CR in children with KD.

Variables	CR-group (*n* = 37)	NCR-group (*n* = 77)	*P*-value	OR	95% CI (OR)
HDL, median (IQR), mmol/L	0.65 (0.50–0.84)	0.77 (0.64–1.01)	**0.022**	0.120	0.020–0.734
LDL, median (IQR), mmol/L	3.00 (2.27–3.46)	2.51 (1.98–3.10)	**0.024**	1.675	1.069–2.623
CYP2C19 LOF allele carriers*, n (%)	29 (78.4%)	34 (44.2%)	**0.005**	3.922	1.504–10.282

CR, clopidogrel resistance; NCR, non-clopidogrel resistance; KD, Kawasaki disease; OR, odds ratio; CI, confidence interval; IQR, interquartile range, P25-P75; HDL, high-density lipoprotein; LDL, low-density lipoprotein; LOF, loss-of-function.

*CYP2C19 LOF allele carriers were defined as patients with at least one LOF allele (*1/*2, *1/*3, *2/*2, *2/*3, *3/*3, *2/*17, or *3/*17). Bold values represent P values less than 0.05.

**TABLE 5:** The cut-off values of prediction CR with CYP2C19 LOF, HDL, and LDL.

	AUC	SE	95%CI	Sensitivity	Specificity	PPV	NPV	*P*-value
HDL ≤ 0.70 mmol/L	0.664	0.056	0.57–0.75	64.9%	67.5%	49.0%	80.0%	0.003
LDL ≥ 2.92 mmol/L	0.630	0.056	0.54–0.72	62.2%	68.8%	48.9%	79.1%	0.019
CYP2C19 LOF allele (+)*	0.671	0.045	0.58–0.76	78.4%	55.8%	46.0%	84.3%	< 0.001

CR, clopidogrel resistance; CI, confidence interval; HDL, high-density lipoprotein; LDL, low-density lipoprotein; LOF, loss-of-function.

*Patients with at least one LOF allele (*1/*2, *1/*3, *2/*2, *2/*3, *3/*3, *2/*17, or *3/*17); NPV, negative predictive value; PPV, positive predictive value.

## Discussion

In this study, the incidence of CR was 31.4% in children with KD, which was higher than that in adults with acute coronary syndrome (ACS) and/or undergoing percutaneous coronary intervention (PCI) (18, 24). The results also showed that carrying the CYP2C19 LOF allele, low levels of HDL, and high levels of LDL were independently associated with CR. The sensitivity and specificity of the combination of the above indicators for predicting CR in children with KD were 70.3 and 74.0%, respectively.

Clopidogrel, an inactive prodrug, needs to be converted to its active metabolite by hepatic cytochrome P450 (CYP) enzymes to exert its antiplatelet effect, and CYP2C19 is the most important enzyme involved in clopidogrel metabolism (13). Previous studies have identified the possession of CYP2C19 LOF alleles as an independent risk factor for CR, because it lowers the conversion rates of clopidogrel by the CYP2C19 enzyme (25). Randomized clinical trial and real-world implementation data also have demonstrated that CYP2C19 genotype-guided selection is expected to improve efficacy and reduce adverse effects of antiplatelet therapy in adults (26, 27). However, there is a lack of clinical data in the pediatric population (16). The frequency of CYP2C19 LOF alleles in the present study of Chinese KD patients was 56.9%, which was similar to previous data in other East Asian populations (approximately 60%) and higher than data in other populations (approximately 30% in African American and Caucasian populations) (28–30). Furthermore, this study found that the possession of CYP2C19 LOF alleles was an independent risk factor for CR in Chinese KD children, which was consistent with studies in adults (25).

In this study, it was found that the patients’s phenotypes were not matched to the genotypes, that is, some KD patients carrying CYP2C19 LOF allele still presented with NCR. The possible mechanisms are as follows, existing studies strongly suggest that insufficient plasma concentrations of the active metabolite of clopidogrel resulted in CR. Low levels of the active metabolite of clopidogrel might be resulted from limited intestinal absorption, which may be affected by ABCB1 gene polymorphism; functional variability in P450 isoenzyme activity influenced by drug-drug interactions as well as other clinical factors; and the genetic variations in CYP450 isoenzymes genes (CYP2C19, CYP1A2, CYP2B6, CYP2C9, and CYP3A4) (9, 13). Therefore, carrying only the CYP2C19 LOF allele is not equivalent to CR. Carrying the CYP2C19 LOF allele is one of factors for poor metabolism of clopidogrel. It is a complex process, may be affected by other gene polymorphisms and a variety of complex clinical factors. In order to research the clinical efficacy of clopidogrel more accurately, plasma concentrations of the active metabolite of clopidogrel will be measured in the future studies.

As a prodrug, clopidogrel is absorbed through the intestine, metabolized in the liver, and excreted to urine (50%) or feces (46%) (31, 32). After absorption in the intestine, approximately 85% of clopidogrel is transformed by hepatic carboxylesterase 1 into an inactive carboxylic acid while 15% is available to undergo hepatic metabolism mediated by CYP450 enzyme (especially the CYP2C19) to yield the active metabolite (31, 32). Therefore, in addition to genetic factors, clinical factors such as clopidogrel dosage, platelet function, liver metabolic function, and renal function can also affect the antiplatelet effect of clopidogrel (10, 13, 18–20). In this study, there was no difference in the dosage of clopidogrel between the different response groups of KD children treated with clopidogrel, thus excluding the effect of clopidogrel dosage on CR. We further comprehensively analyzed the remaining laboratory indicators on the effectiveness of clopidogrel in children with KD, and found that low levels of HDL and high levels of LDL were independent risk factors for the occurrence of CR in children with KD in China. Our result aligned with a study in adults with coronary artery disease (CAD), which demonstrated that high levels of LDL and low levels of HDL were correlated with low response to clopidogrel (33).

Lipoproteins can influence platelet count and characteristics and modulate the risk of atherothrombosis via the megakaryocyte-platelet hemostatic axis (34). In the present study it was found that HDL was a protective factor for the occurrence of CR in children with KD. Our finding was in line with a previous study of Wadowski et al. suggesting that low HDL levels were linked to impaired clopidogrel-mediated platelet inhibition after angioplasty (35). Similarly, another study found that low HDL levels were associated with CR in CAD patients (36). Moreover, it was shown that patients with low HDL levels might be more prone to platelet hyperreactivity in the period after clopidogrel cessation (34), and the infusion of reconstituted HDL was highly effective in inhibiting platelet hyperactivity in diabetic patients (37). However, a study of ACS patients undergoing PCI suggested that HDL was inversely associated with platelet activation parameters but not with clopidogrel response variability (38). Although the specific mechanism of the effect of HDL on the antiplatelet effect of clopidogrel is still unclear, it is generally considered that in addition to its role in reverse cholesterol transport, HDL also inhibits platelet activation *in vivo* and *in vitro* to mediate antithrombotic effects (39). Studies have shown that HDL can prevent platelet hyperreactivity by limiting the excessive accumulation of cholesterol in platelet membranes and regulating platelet signaling pathways by binding specific receptors on the platelet surface (34, 39). Meanwhile, HDL also stimulates the endothelial production of nitric oxide and prostacyclin, which are potent inhibitors of platelet activation (39). Altogether, HDL may inhibit platelet function through various pathways, thereby reducing the occurrence of CR.

Studies have shown that LDL promotes platelet activation and platelet-dependent thrombus formation (40). in the study it was found that LDL was an independent risk factor for CR in children with KD, which was consistent with the conclusion of Bobescu et al. that elevated LDL levels significantly increased the occurrence of CR in CAD patients (33). Previous studies showed that LDL augmented platelet activation *in vitro* and *in vivo* (40, 41). There are specific lipoprotein binding sites on the surface of platelets, and LDL, especially oxidized LDL, can specifically bind to them to activate platelets (42). Additionally, LDL can also act on the phospholipase A2 pathway to stimulate platelet release and induce platelet aggregation (40, 42). In brief, high levels of LDL may induce platelet activation, leading to decreased antiplatelet function, which may induce the occurrence of CR.

Our study has several limitations. First, this study was performed at a single center, the sample size was small, lacking multi-center data verification. Second, due to the absence of long-term follow-up, we cannot evaluate the impact of the CR on the long-term complications of coronary artery in children with KD. Finally, the population in the study only featured Chinese children, whether these results generalize to other ethnicities may require further investigation and validation.

## Conclusion

In conclusion, CR was found in children with KD and its incidence was 31.4%. Carrying CYP2C19 LOF allele, low levels of HDL, and high levels of LDL were independent risk factors for CR in children with KD. These indicators may serve as valuable biomarkers for predicting CR in children with KD and further guide the pediatricians to choose individualized antiplatelet therapy.

## Data availability statement

The original contributions presented in the study are included in the article/supplementary material, further inquiries can be directed to the corresponding author/s.

## Ethics statement

The studies involving human participants were reviewed and approved by the Ethics Committee of the Capital Institute of Pediatrics (No. SHERLL 2018020). Written informed consent to participate in this study was provided by the participants’ legal guardian/next of kin.

## Author contributions

XL conceptualized, designed the study, contributed to the interpretation of the results, reviewed, and revised the manuscript. LM and MZ completed the data analyses and drafted the manuscript. YC contributed to the revision of the manuscript. LS contributed important intellectual content. All authors agreed with the submission of this version of the manuscript.

## References

[1] Peng H Wu Z Liu Y Li L Kong S Wu J Low-dose antithrombotic treatment in coronary thrombosis of Kawasaki disease. *Pediatr Cardiol.* (2015) 36:503–8. 10.1007/s00246-014-1040-1 25298222

[2] Dionne A Dahdah N Singh-Grewal D Burgner DP Newburger JW de Ferranti SD. Anti-thrombosis management of patients with Kawasaki disease: results from an international survey. *Int J Cardiol.* (2020) 307:154–8. 10.1016/j.ijcard.2019.10.045 31753581

[3] McCrindle BW Rowley AH Newburger JW Burns JC Bolger AF Gewitz M Diagnosis, treatment, and long-term management of Kawasaki disease: a scientific statement for health professionals from the American heart association. *Circulation.* (2017) 135:e927–99. 10.1161/CIR.0000000000000484 28356445

[4] Fukazawa R Kobayashi J Ayusawa M Hamada H Miura M Mitani Y Jcs/Jscs 2020 guideline on diagnosis and management of cardiovascular sequelae in Kawasaki disease. *Circ J.* (2020) 84:1348–407. 10.1253/circj.CJ-19-1094 32641591

[5] Committee CS. A randomised, blinded, trial of clopidogrel versus aspirin in patients at risk of ischaemic events (Caprie). Caprie steering committee. *Lancet.* (1996) 348:1329–39. 10.1016/s0140-6736(96)09457-3 8918275

[6] Diener HC Bogousslavsky J Brass LM Cimminiello C Csiba L Kaste M Aspirin and clopidogrel compared with clopidogrel alone after recent ischaemic stroke or transient ischaemic attack in high-risk patients (Match): randomised, double-blind, placebo-controlled trial. *Lancet.* (2004) 364:331–7. 10.1016/S0140-6736(04)16721-4 15276392

[7] Sabatine MS Cannon CP Gibson CM Lopez-Sendon JL Montalescot G Theroux P Effect of clopidogrel pretreatment before percutaneous coronary intervention in patients with st-elevation myocardial infarction treated with fibrinolytics: the pci-clarity study. *JAMA.* (2005) 294:1224–32. 10.1001/jama.294.10.1224 16143698

[8] Wang Y Wang Y Zhao X Liu L Wang D Wang C Clopidogrel with aspirin in acute minor stroke or transient ischemic attack. *N Engl J Med.* (2013) 369:11–9. 10.1056/NEJMoa1215340 23803136

[9] Patti G Micieli G Cimminiello C Bolognese L. The role of clopidogrel in 2020: a reappraisal. *Cardiovasc Ther.* (2020) 2020:8703627. 10.1155/2020/8703627 32284734 PMC7140149

[10] Wisniewski A Filipska K. The phenomenon of clopidogrel high on-treatment platelet reactivity in ischemic stroke subjects: a comprehensive review. *Int J Mol Sci.* (2020) 21:6408. 10.3390/ijms21176408 32899176 PMC7503235

[11] Guirgis M Thompson P Jansen S. Review of aspirin and clopidogrel resistance in peripheral arterial disease. *J Vasc Surg.* (2017) 66:1576–86. 10.1016/j.jvs.2017.07.065 28893489

[12] Bonello L Tantry US Marcucci R Blindt R Angiolillo DJ Becker R Consensus and future directions on the definition of high on-treatment platelet reactivity to adenosine diphosphate. *J Am Coll Cardiol.* (2010) 56:919–33. 10.1016/j.jacc.2010.04.047 20828644

[13] Akkaif MA Daud NAA Sha’aban A Ng ML Abdul Kader MAS Noor DAM The role of genetic polymorphism and other factors on clopidogrel resistance (Cr) in an Asian population with coronary heart disease (Chd). *Molecules.* (2021) 26:1987. 10.3390/molecules26071987 33915807 PMC8036376

[14] Pereira NL Rihal CS So DYF Rosenberg Y Lennon RJ Mathew V Clopidogrel pharmacogenetics. *Circ Cardiovasc Interv.* (2019) 12:e007811. 10.1161/CIRCINTERVENTIONS.119.007811 30998396 PMC6581205

[15] Scott SA Sangkuhl K Stein CM Hulot JS Mega JL Roden DM Clinical pharmacogenetics implementation consortium guidelines for Cyp2c19 genotype and clopidogrel therapy: 2013 update. *Clin Pharmacol Ther.* (2013) 94:317–23. 10.1038/clpt.2013.105 23698643 PMC3748366

[16] Lee CR Luzum JA Sangkuhl K Gammal RS Sabatine MS Stein CM Clinical pharmacogenetics implementation consortium guideline for Cyp2c19 genotype and clopidogrel therapy: 2022 update. *Clin Pharmacol Ther.* (2022): 10.1002/cpt.2526 [Epub ahead of print]. 35034351 PMC9287492

[17] Sibbing D Koch W Gebhard D Schuster T Braun S Stegherr J Cytochrome 2c19*17 allelic variant, platelet aggregation, bleeding events, and stent thrombosis in clopidogrel-treated patients with coronary stent placement. *Circulation.* (2010) 121:512–8. 10.1161/CIRCULATIONAHA.109.885194 20083681

[18] Warlo EMK Arnesen H Seljeflot I. A brief review on resistance to P2y12 receptor antagonism in coronary artery disease. *Thromb J.* (2019) 17:11. 10.1186/s12959-019-0197-5 31198410 PMC6558673

[19] Wang H Qi J Li Y Tang Y Li C Li J Pharmacodynamics and pharmacokinetics of ticagrelor Vs. clopidogrel in patients with acute coronary syndromes and chronic kidney disease. *Br J Clin Pharmacol.* (2018) 84:88–96. 10.1111/bcp.13436 28921624 PMC5736840

[20] Gremmel T Mueller M Koppensteiner R Panzer S. Liver function is associated with response to clopidogrel therapy in patients undergoing angioplasty and stenting. *Angiology.* (2016) 67:835–9. 10.1177/0003319715609011 26416820

[21] Gentilomo C Huang YS Raffini L. Significant increase in clopidogrel use across U.S. Children’s hospitals. *Pediatr Cardiol.* (2011) 32:167–75. 10.1007/s00246-010-9836-0 21132568

[22] Huang JY Soeharto DA Damara I Ignjatovic V Linden MD Monagle P. Antiplatelet therapy monitoring in neonates and children. *Semin Thromb Hemost.* (2019) 45:73–85. 10.1055/s-0038-1676375 30566968

[23] Bliden KP DiChiara J Tantry US Bassi AK Chaganti SK Gurbel PA. Increased risk in patients with high platelet aggregation receiving chronic clopidogrel therapy undergoing percutaneous coronary intervention: is the current antiplatelet therapy adequate? *J Am Coll Cardiol.* (2007) 49:657–66. 10.1016/j.jacc.2006.10.050 17291930

[24] Su Q Li J Tang Z Yang S Xing G Liu T Association of Cyp2c19 polymorphism with clopidogrel resistance in patients with acute coronary syndrome in China. *Med Sci Monit.* (2019) 25:7138–48. 10.12659/MSM.915971 31543510 PMC6775793

[25] Peng W Shi X Xu X Lin Y. Both Cyp2c19 and Pon1 Q192r genotypes influence platelet response to clopidogrel by thrombelastography in patients with acute coronary syndrome. *Cardiovasc Ther.* (2019) 2019:3470145. 10.1155/2019/3470145 31772608 PMC6739775

[26] Notarangelo FM Maglietta G Bevilacqua P Cereda M Merlini PA Villani GQ Pharmacogenomic approach to selecting antiplatelet therapy in patients with acute coronary syndromes: the pharmclo trial. *J Am Coll Cardiol.* (2018) 71:1869–77. 10.1016/j.jacc.2018.02.029 29540324

[27] Zhang Y Shi XJ Peng WX Han JL Lin BD Zhang R Impact of implementing Cyp2c19 genotype-guided antiplatelet therapy on P2y12 inhibitor selection and clinical outcomes in acute coronary syndrome patients after percutaneous coronary intervention: a real-world study in China. *Front Pharmacol.* (2020) 11:582929. 10.3389/fphar.2020.582929 33551797 PMC7854467

[28] Wang Y Zhao X Lin J Li H Johnston SC Lin Y Association between Cyp2c19 loss-of-function allele status and efficacy of clopidogrel for risk reduction among patients with minor stroke or transient ischemic attack. *JAMA.* (2016) 316:70–8. 10.1001/jama.2016.8662 27348249

[29] He L Chen S Li J Xie X Huang L Kuang Y Genetic and phenotypic frequency distribution of Cyp2c9, Cyp2c19 and Cyp2d6 in over 3200 Han Chinese. *Clin Exp Pharmacol Physiol.* (2020) 47:1659–63. 10.1111/1440-1681.13357 32469422

[30] Jeong YH. "East Asian Paradox": challenge for the current antiplatelet strategy of “one-guideline-fits-all races” in acute coronary syndrome. *Curr Cardiol Rep.* (2014) 16:485. 10.1007/s11886-014-0485-4 24668607

[31] Jiang XL Samant S Lesko LJ Schmidt S. Clinical pharmacokinetics and pharmacodynamics of clopidogrel. *Clin Pharmacokinet.* (2015) 54:147–66. 10.1007/s40262-014-0230-6 25559342 PMC5677184

[32] Farid NA Kurihara A Wrighton SA. Metabolism and disposition of the thienopyridine antiplatelet drugs ticlopidine, clopidogrel, and prasugrel in humans. *J Clin Pharmacol.* (2010) 50:126–42. 10.1177/0091270009343005 19948947

[33] Bobescu E Covaciu A Rus H Rogozea LM Badea M Marceanu LG. Low response to clopidogrel in coronary artery disease. *Am J Ther.* (2020) 27:e133–41. 10.1097/MJT.0000000000001099 31688068

[34] Dukanovic N Obradovic S Zdravkovic M Durasevic S Stojkovic M Tosti T Lipids and antiplatelet therapy: important considerations and future perspectives. *Int J Mol Sci.* (2021) 22:3180. 10.3390/ijms22063180 33804754 PMC8003871

[35] Wadowski PP Lee S Kopp CW Koppensteiner R Panzer S Gremmel T. Low levels of high-density lipoprotein cholesterol are linked to impaired clopidogrel-mediated platelet inhibition. *Angiology.* (2018) 69:786–94. 10.1177/0003319718760074 29482349

[36] Al-Azzam SI Alzoubi KH Khabour OF Nusair MB Al-Hadidi H Awidi A Factors that contribute to clopidogrel resistance in cardiovascular disease patients: environmental and genetic approach. *Int J Clin Pharmacol Ther.* (2013) 51:179–86. 10.5414/CP201784 23357840

[37] Calkin AC Drew BG Ono A Duffy SJ Gordon MV Schoenwaelder SM Reconstituted high-density lipoprotein attenuates platelet function in individuals with type 2 diabetes mellitus by promoting cholesterol efflux. *Circulation.* (2009) 120:2095–104. 10.1161/CIRCULATIONAHA.109.870709 19901191

[38] Tselepis AD Tsoumani ME Kalantzi KI Dimitriou AA Tellis CC Goudevenos IA. Influence of high-density lipoprotein and paraoxonase-1 on platelet reactivity in patients with acute coronary syndromes receiving clopidogrel therapy. *J Thromb Haemost.* (2011) 9:2371–8. 10.1111/j.1538-7836.2011.04541.x 22008470

[39] van der Stoep M Korporaal SJ Van Eck M. High-density lipoprotein as a modulator of platelet and coagulation responses. *Cardiovasc Res.* (2014) 103:362–71. 10.1093/cvr/cvu137 24891399

[40] Chatterjee M Rath D Schlotterbeck J Rheinlaender J Walker-Allgaier B Alnaggar N Regulation of oxidized platelet lipidome: implications for coronary artery disease. *Eur Heart J.* (2017) 38:1993–2005. 10.1093/eurheartj/ehx146 28431006

[41] Petersen-Uribe A Kremser M Rohlfing AK Castor T Kolb K Dicenta V Platelet-derived Pcsk9 is associated with Ldl metabolism and modulates atherothrombotic mechanisms in coronary artery disease. *Int J Mol Sci.* (2021) 22:11179. 10.3390/ijms222011179 34681838 PMC8538687

[42] Gasecka A Rogula S Szarpak L Filipiak KJ. Ldl-cholesterol and platelets: insights into their interactions in atherosclerosis. *Life (Basel).* (2021) 11:39. 10.3390/life11010039 33440673 PMC7826814

